# Environmental Exposures around Conception: Developmental Pathways Leading to Lifetime Disease Risk

**DOI:** 10.3390/ijerph18179380

**Published:** 2021-09-06

**Authors:** Tom P. Fleming, Congshan Sun, Oleg Denisenko, Laura Caetano, Anan Aljahdali, Joanna M. Gould, Pooja Khurana

**Affiliations:** 1 Biological Sciences, Southampton General Hospital, University of Southampton, Southampton SO16 6YD, UK; laura.cnc.caetano@gmail.com (L.C.); araljahdali@uj.edu.sa (A.A.); poojak@hawaii.edu (P.K.); 2 Departments of Neurology and Neuroscience, Johns Hopkins University School of Medicine, Baltimore, MD 21205, USA; csun23@jhmi.edu; 3 Center for Genetic Muscle Disorders, Hugo W. Moser Research Institute at Kennedy Krieger Institute, Baltimore, MD 21205, USA; 4 Department of Medicine, University of Washington, 850 Republican St., Rm 242, Seattle, WA 98109, USA; odenis@uw.edu; 5 Department of Biological Sciences, Faculty of Science, Alfaisaliah campus, University of Jeddah, Jeddah 23442, Saudi Arabia; 6 Clinical Neurosciences and Psychiatry, Clinical and Experimental Sciences, Faculty of Medicine, University of Southampton, Southampton SO16 6YD, UK; joannagould035@gmail.com; 7 Institute for Biogenesis Research, Research Corporation of the University of Hawaii, Manoa, Honolulu, HI 96822, USA

**Keywords:** DOHaD, peri-conception, blastocyst, maternal undernutrition, low protein diet, trophectoderm, primitive endoderm, uterine fluid, mTORC1, ribosome biogenesis

## Abstract

Environment around conception can influence the developmental programme with lasting effects on gestational and postnatal phenotype and with consequences for adult health and disease risk. Peri-conception exposure comprises a crucial part of the ‘Developmental Origins of Health and Disease’ (DOHaD) concept. In this review, we consider the effects of maternal undernutrition experienced during the peri-conception period in select human models and in a mouse experimental model of protein restriction. Human datasets indicate that macronutrient deprivation around conception affect the epigenome, with enduring effects on cardiometabolic and neurological health. The mouse model, comprising maternal low protein diet exclusively during the peri-conception period, has revealed a stepwise progression in altered developmental programming following induction through maternal metabolite deficiency. This progression includes differential effects in extra-embryonic and embryonic cell lineages and tissues, leading to maladaptation in the growth trajectory and increased chronic disease comorbidities. The timeline embraces an array of mechanisms across nutrient sensing and signalling, cellular, metabolic, epigenetic and physiological processes with a coordinating role for mTORC1 signalling proposed. Early embryos appear active participants in environmental sensing to optimise the developmental programme for survival but with the trade-off of later disease. Similar adverse health outcomes may derive from other peri-conception environmental experiences, including maternal overnutrition, micronutrient availability, pollutant exposure and assisted reproductive treatments (ART) and support the need for preconception health before pregnancy.

## 1. Introduction

The period around conception is recognised not only as the key transition between generations but also a vulnerable time in our gestation to adverse environmental influences. These can alter the course of development and impose lasting effects on health through life. By environment, a wide range of external influences are relevant, including the quality of maternal nutrition, the level of maternal infection and sickness, exposure to environmental toxins and alcohol consumption. Similar paternal characteristics are important, and the varied conditions used in vitro during both clinical and domestic animal assisted reproductive treatment (ART) [1,2]. The environment affects the homeostasis of the gametes and early embryo, impacting many fundamental processes in the developmental programme, influencing embryo proliferation, growth, metabolism, signalling activity and gene expression. Small ‘shifts’ in phenotype mediated through the environment around the time of conception have the capacity to deflect the direction of development, enduring throughout gestation and the lifespan. Peri-conception exposures influence lifetime health and chronic disease risk across cardiometabolic and neurological co-morbidities, a central theme in the concept of Developmental Origins of Health and Disease (DOHaD) [1,2]. Peri-conception DOHaD is evident in human and both small and large domesticated animal models [3,4]. Thus, societal consequences may span from direct clinical health burdens to the efficiency and economics of food production.

The DOHaD hypothesis is substantiated by the epigenetic restructuring of chromatin that occurs across the genome in the early embryo as the embryonic programme is activated to achieve development and replaces that of the parental gametes. Epigenome reorganisation is mediated through extensive remodelling principally of DNA methylation and histone modifications to coordinate gene expression [5,6]. There is growing evidence that disturbance in the epigenetic profile by environmental factors represents a critical causal route towards offspring health outcomes. However, long-term DOHaD effects are further propagated by a range of contributory signalling, cellular, metabolic and physiological mechanisms. Moreover, environmental restructuring from peri-conception exposures may be viewed not only as perturbations but also as conserved adaptive processes to aid survival, but with trade-offs in long-term health.

In this review, we focus on environmental exposure mediated through maternal undernutrition occurring around conception, which has been shown to increase risk of offspring adult disease. Long-term health effects mediated through paternal undernutrition around conception are covered elsewhere [1,2,7,8]. Nutrition-related factors remain a major burden in terms of global health, accounting for 45% of deaths in children under five. Undernutrition was reported in 2018 to cause some 200 million children aged under five to suffer from stunting or wasting and at least 340 million from hidden hunger [9]. However, understanding of causal relationships between macronutrient provision during pregnancy and child health remain limited. We review evidence from select human datasets as well as controlled animal models including an in-depth review of our mouse maternal low protein diet (LPD) model. We consider how nutritional quality may interface with the early embryo and how the embryo may respond in ways that persist beyond the peri-conception period, involving diverse cell lineages and multiple mechanisms, and representing a stepwise progression from altered cellular homeostasis to adult maladaptation and disease risk. In this context, we propose a key role for nutritional signalling pathways in coordinating long-term consequences. We then briefly consider how other conditions comprising maternal overnutrition, micronutrient availability, pollutant exposure and ART may lead to similar long-term health risks. Lastly, we evaluate how these biological processes impact on policy for maternal intervention in pregnancy and the case for preconception care to promote offspring health.

## 2. Human Peri-Conception Undernutrition and Offspring Health

A well-researched and clinically relevant example of the effect of poor maternal nutrition around conception relates to the Dutch Hunger Winter (DHW) in 1944–1945 during World War 2. The population in the western region of the Netherlands was subjected to severe food restriction, below 1000 calories/day, comprising mainly potato, bread and sugar-beet, imposed by the German occupiers for a 5-month period. The famine was applied and later relieved at precise times and medical records were maintained throughout and afterwards. This has enabled epidemiologists to research the health consequences over the lifetime of offspring from affected pregnant mothers. Most critical, given the precision of famine duration, was the ability to probe the effects of famine experienced at early, middle or later stages of pregnancy, perceptively reviewed recently [10].

Whilst exposure to the DHW famine in utero at any time during gestation generated a poorer health prognosis in adulthood versus control cohorts born either before or after the famine, there is compelling evidence that early exposure including the conception period led to increased adult disease risk. This has been reported across metabolic capacity, such as glucose tolerance and insulin regulation [11], risk of adult obesity [12], increased plasma lipid levels [13], increased and earlier occurrence of coronary heart disease [14,15] and adult mortality [16,17]. Moreover, neurological disorders were increased in famine exposure in early gestation, including schizophrenia [18], poorer cognitive function [19], smaller brain volume [20] and premature brain ageing [21]. The extensive dataset from the DHW is probably unique in the scope of findings but other famines, such as the Chinese Great Leap Forward (1959–61), have also demonstrated the enhanced risk to offspring health mediated through exposure during early gestation on adult hypertension [22] and incidence of schizophrenia [23]. From the wealth of data published from the DHW, there is also ample evidence that sex differences exist in the phenotype of affected offspring with women more prone to obesity and cardiovascular outcomes, while men were more vulnerable to brain effects and mental disorders [10].

From a causative perspective, there is also growing evidence that the genome of DHW-exposed offspring is altered epigenetically compared with that of controls where pregnancy was outside the famine period. Reduced DNA methylation of the imprinted *IGF2* locus was identified in late adulthood in famine survivors exposed during conception [24]. Genome-wide screening has revealed differential methylation of expression pathways regulating growth and metabolism in particular in those exposed to the famine during early gestation [25,26,27]. Similar epigenetic relationships have also been found in adults exposed to the Chinese famine [28,29].

The close association between maternal diet at conception and offspring postnatal health has also been recognised in an extensive nutritional study in the Gambia, where seasonal variation in maternal nutrient intake due to previous harvest depletion and loss of caloric intake associates with the risk of low birthweight, later-life disease and mortality [30]. We see here again an epigenetic association with seasonal health outcomes. Thus, in individuals who were conceived in the nutritionally challenged season, DNA methylation was increased within select regions of the epigenome that exhibited stochastic, interindividual variation, known as metastable epialleles (MEs). Moreover, the epigenetic changes occurred across all three germ layers, indicating a pre-gastrulation origin, and persisted through to adulthood, providing good evidence that maternal diet at conception can alter the epigenome of the embryo in an enduring manner [31,32]. Specific ME targets investigated in this model include the methylation pattern of the imprinted gene *VTRNA2-1*, established before implantation and which remained stable through to adulthood [33]. Screening of human peri-conception embryo samples has identified many MEs with enrichment for proximal enhancers and transcription start sites [34]. In a broader context, season of conception may be associated with many features of the human phenotype and disease risk in later life [35].

Whether and how the altered ME methylation pattern may function in regulating phenotype in offspring is the central question moving forwards, but a strategy to identify associations with gene pathways is feasible, despite the variation in human genotype and other confounders. One mechanism linking the seasonal nature of maternal nutrition at conception in the Gambia with offspring phenotype is telomere length in childhood [36]. Telomere shortening is associated with many chronic cardiometabolic diseases [37] and is, thus, an interesting line of enquiry.

## 3. Mouse Peri-Conception Undernutrition and Offspring Health

Substantial progress is being made in unravelling the mechanistic basis of human disease origin from peri-conceptional undernutrition through the epidemiologic population studies and the pursuit of epigenetic pathways as described above. However, the inter-generational nature of the relationship needs more detailed consideration of the time course and stepwise pattern of cause and effect to link embryo environment with adult offspring health. Animal models with controlled genotype, precise experimental design and shorter lifespan reduce confounders and permit insightful understanding of this timeline and origin of disease. Indeed, animal models reveal not just epigenetic mechanisms, but physiological, signalling and cellular processes active in setting lifetime health from peri-conceptional experience.

**Figure 1:**
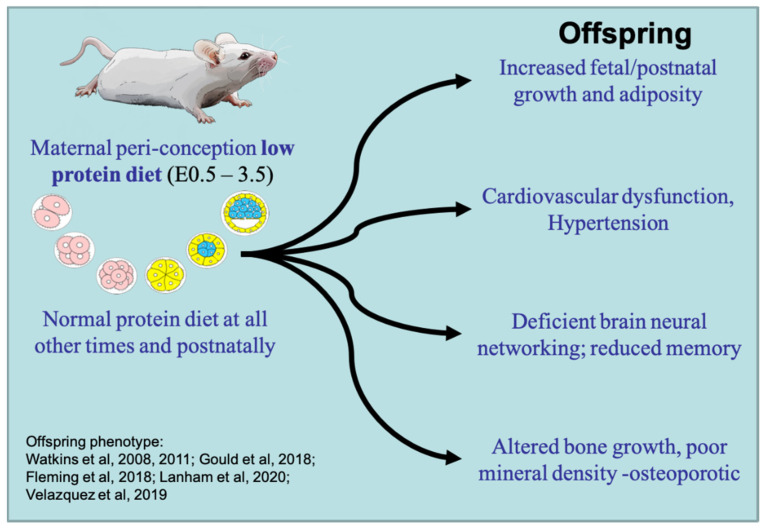
Diagrammatic representation of the maternal Emb-LPD mouse model. Maternal low protein diet is provided exclusively for the preimplantation period with normal nutrition, thereafter leading to co-morbidities affecting growth, cardiometabolic, neurological and skeletal health. Key references for these outcomes are identified, in sequence being 40, 42, 43, 1, 44, 2. See text for details.

We have made extensive use of a mouse maternal protein-deficient diet fed either throughout gestation (low protein diet, 50% reduction but isocaloric, LPD; see [38] for diet composition) or exclusively during the period of preimplantation development (E1.5–3.5), with normal nutrition at other times and postnatally (Emb-LPD). This model allows for the investigation of embryo induction and immediate and longer-term responses to maternal diet through to disease phenotype (reviewed previously [1,39]). The Emb-LPD protocol (compared with control, normal protein diet, NPD) is sufficient to enhance the risk of comorbidities across growth, cardiometabolic, neurological and skeletal criteria in offspring (Figure 1). Key characteristics mediated through maternal Emb-LPD in offspring include relative hypertension with reduced capacity for arterial dilatation [40,41]; an increased foetal and postnatal growth trajectory with excess adiposity [40,42]; and reduced survival of neural stem cells and altered neural networking capacity in the foetal brain, leading to dysfunctional adult behaviour affecting locomotor and memory performance [40,43]. In a recent study, we have found the increased foetal growth trajectory to adversely affect skeletal development with Emb-LPD, causing increased foetal bone formation proportionate to foetal weight across the central and peripheral skeleton, especially in males. However, micro-3D-computed tomography revealed that the increased bone growth in late gestation was of reduced mineral density compared with controls [44], a characteristic recognised as vulnerable to osteoporotic disease in adulthood [45].

**Figure 2:**
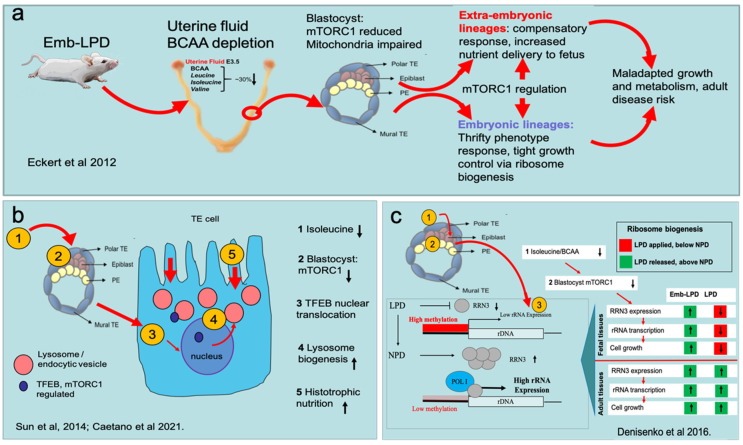
Diagrammatic representation of the stepwise nature of altered developmental programme mediated through maternal Emb-LPD. (**a**) Overview, from BCAA (branched-chain amino acid) depletion in uterine fluid, reducing mTORC1 signalling in the blastocyst, leading to distinct changes in extra-embryonic and embryonic tissues and later-life disease. (**b**) Activation of increased histotrophic nutrition in trophectoderm (TE) mediated through low BCAA availability and TFEB translocation. A similar mechanism occurs in the primitive endoderm (PE) derivatives. Orange numbers represent steps in induction of histotrophic nutrition listed on the right where black arrows indicate increased or decreased levels/activity. (**c**) Activation of altered ribosome biogenesis in somatic tissues (derived from Epiblast) mediated through dietary sensing, RRN3 expression and rRNA transcription. Orange numbers represent steps in induction listed on the centre/right and, for 3, the effect of diet on ribosome biogenesis (bottom left) and life stage (bottom right). Key references for these outcomes are identified; in sequence being (**a**) 46; (**b**) 63 and 65; (**c**) 94. See text for details.

These comorbidities following maternal Emb-LPD, as in the human disease models, affected either both sexes (cardiovascular) or mainly, but not exclusively, one sex (females: growth/adiposity, behaviour; males: bone structure). A similar phenotype occurred in response to LPD throughout gestation, but pertinent distinctions are referred to below. The pathway from maternal dietary environment around conception to disease manifestation in adult offspring is multifactorial and sequential (Figure 2a). First, we consider the induction mechanism, how the diet alters maternal metabolism, the immediate changing environment experienced by embryos in the reproductive tract before implantation and how this interface modifies embryo phenotype and homeostasis.

### 3.1. Embryo Induction of Altered Developmental Programming by Maternal Undernutrition

The maternal systemic environment following Emb-LPD becomes deficient in several amino acids (AAs) and mildly hyperglycaemic (raised glucose, reduced insulin) during the period of preimplantation development [46]. A similar maternal physiological condition occurred in our earlier rat Emb-LPD model [38] and, in an independent study, altered AA metabolism occurred in preovulatory mouse oocytes in response to maternal LPD [47].

These maternal physiological outcomes create environmental conditions that in turn influence embryo physiological potential. AAs perform biosynthetic, metabolic, protective and signalling roles in early embryogenesis [48,49,50] and AA turnover by human embryos is associated with their viability [51,52]. Thus, AA deficiency in vivo may influence embryo phenotype in several ways. However, closer scrutiny of the maternal dietary effect on embryo potential was gained by biochemical analysis of the AA composition in the immediate uterine fluid (UF) micro-environment experienced by preimplantation embryos during blastocyst morphogenesis (E3.5). UF composition is highly regulated during the peri-conception period to coordinate embryo morphogenesis and implantation potential across species [53,54]. Generally, AA concentrations increase in mouse UF over the full preimplantation period [55], but we have found maternal diet to impose selective effects on signalling AA concentrations. Thus, whilst several AAs, including the branched-chain AAs (BCAAs; leucine, isoleucine, valine), were reduced in concentration in maternal serum by Emb-LPD at the time of blastocyst formation (E3.5), micro-sampling and high-performance liquid chromatography analysis of UF revealed only the BCAAs were significantly depleted (~25–30%) [46]. Moreover, BCAA deficiency in maternal serum following Emb-LPD has also been shown in our rat model [38] and independently by others in both mice and rats at the time of blastocyst morphogenesis in LPD-fed dams [56].

How might BCAA deficiency in the UF in which embryos are ‘bathed’ before implantation affect their physiology? Availability of BCAAs, and leucine in particular, are critical alongside insulin in the activation of the nutrient-sensing signalling pathway to regulate cellular growth and biosynthesis through the mammalian target of rapamycin complex 1 (mTORC1) [57]. Embryos are equipped with AA transporters to regulate BCAA uptake and release and mTORC1 signalling [58,59]; hence, BCAA deficiency may be ‘sensed’ by blastocysts in this micro-environment before implantation. Indeed, quantification of mTORC1 signalling activity in Emb-LPD blastocysts revealed a significant reduction in the phosphorylation of the downstream effector ribosomal S6 [46], which has a critical role in coordinating nutrient availability with cell growth [60] and transcription of factors required for ribosome biogenesis [61].

We consider the reduced mTORC1 signalling activity in blastocysts mediated through depleted BCAA and insulin availability to be the key step in the induction of adverse developmental programming by maternal protein restriction (summarised in Figure 2a). Further support for this comes from an in vitro model where embryo culture in medium with 50% deficiency in BCAAs and insulin (compared with concentrations found in control NPD dams) lead to offspring after transfer with growth and cardiovascular morbidities similar to Emb-LPD offspring [62]. Moreover, this culture environment was sufficient to cause some of the early responses found in Emb-LPD blastocysts and subsequent extra-embryonic cell lineages, discussed below [63,64,65]. However, whilst an mTORC1-mediated induction of developmental reprogramming in blastocysts is attractive and substantiated, it may work alongside other metabolic factors in a multifaceted process. Two further mechanisms mediated through altered AA homeostasis are considered below.

First, maternal and UF threonine levels were reduced by Emb-LPD, but not significantly, only to trend (*p* < 0.1) level [46]. Similarly, reduced threonine was observed in maternal serum following LPD in our rat study [38] and independently in both mouse and rat LPD models at the blastocyst stage [56]. Threonine facilitates homocysteine (Hcy) re-methylation to methionine in the transmethylation cycle of 1-carbon metabolism [56,66]. Indeed, Hcy is significantly elevated in LPD maternal serum [56] which may reflect insufficient threonine availability or deficient capacity for its enzymatic dissipation [66]. Moreover, the methionine level was increased by Emb-LPD in both UF at E4.5 and within blastocysts [46]. Thus, an additional mediator in adverse blastocyst developmental programming in response to LPD may be direct effects on the cycling of sulphur-containing amino acids affecting 1-carbon metabolism, DNA methylation and epigenetic regulation [67,68].

Second, lysine is reduced in Emb-LPD maternal serum and in blastocysts at E3.5, and also, at trend level, in the UF [46]. Lysine may act as a source of glutamate production and the maintenance of signalling, pluripotency and proliferation in embryonic stem cells, and thus, may also be critical in induction of adverse developmental programming [69]. However, we consider this pathway to be less likely since glutamic acid levels are unchanged in Emb-LPD serum and UF and, indeed, are increased in Emb-LPD blastocysts [46].

In addition to AA-related changes in blastocyst signalling activity and phenotype mediated through Emb-LPD, more direct metabolic changes have been reported. Maternal LPD during 3–4 weeks before mating and during preimplantation development led to an altered cellular distribution of mitochondria coupled with a reduction in membrane potential and increased mitochondrial calcium levels [70]. This suggests a reduction in energy availability, although embryo ATP levels and reactive oxygen species (ROS) were not altered by the maternal diet in this study [70]. Altered AA metabolism and an impaired mitochondrial phenotype has also been shown following rat maternal LPD in preovulatory oocytes with increased abnormal mitochondrial ultrastructure and changed expression of enzymes regulating mitochondrial function [47]. Similarly, in human trophoblast cells, AA starvation adversely affected mitochondrial morphology with increased stress-induced mitochondrial hyperfusion [71].

AA sensing following Emb-LPD through reduced mTORC1 signalling and S6 phosphorylation may also network across other cellular regulatory processes. Thus, sensing of AAs leading to S6 phosphorylation status also involves p38 or ERK mitogen-activated protein kinase (MAPK) signalling [72]. MAPK is a highly conserved network responsive to environmental stimuli, which may regulate diverse cellular activities such as proliferation, differentiation or apoptosis through control of target genes [73,74]. In early embryos, MAPK signalling contributes to pluripotency and differentiation, especially of the primitive endoderm (PE) extraembryonic lineage [75,76,77]. MAPK signalling also appears to have a role in response to AA deprivation in mouse blastocysts by inducing anti-oxidant gene expression to counteract oxidative stress, thereby protecting embryo viability [64].

The induction mechanism(s) in the early embryo leads to altered programming of both the extra-embryonic and embryonic cell lineages formed during blastocyst morphogenesis and beyond. These responses to the sensing of maternal metabolites are key for the propagation of peri-conception events through gestation and the acquisition of physiological maladaptation that associates with later life disease and are reviewed in the next sections. We see a continued importance in mTORC1 signalling and downstream consequences during this timeline.

### 3.2. Blastocyst Nutrient Sensing Activates Compensatory Responses in Extra-Embryonic Lineages

The blastocyst outer cell layer, the trophectoderm (TE), generates the embryonic portion of the chorio-allantoic placenta, while the primitive endoderm (PE), which forms on the blastocoelic face of the inner cell mass (ICM), is the progenitor of the visceral yolk sac, which also supports maternal nutrient provision, especially prior to the functioning of the placenta [78,79,80]. In both extra-embryonic lineages, maternal Emb-LPD/LPD leads to a series of phenotypic changes that collectively appear compensatory, enhancing the capacity for maternal nutrient delivery to combat the restricted nature of the maternal diet (summarised in Figure 2b).

The TE in response to Emb-LPD displays a small increase in proliferation and, in outgrowths, representing an in vitro model for motility and invasiveness associated with the endometrial implantation process, undergoes increased spreading [46]. TE motility at implantation has been shown to be regulated through AAs and mTORC1 activity [81]. A critical change in TE phenotype prior to implantation in response to maternal LPD is increased histotrophic nutrition, the capacity to endocytose and digest the contents of the uterine fluid from apical membrane by fluid-phase and receptor-mediated routes involving multi-ligand megalin and cubilin receptors [82,83,84]. Emb-LPD blastocyst TE cells exhibit increased numbers and collective volume of endocytic and lysosome vesicles with increased megalin presence in the apical cytoplasm and membrane (Figure 2b) [63,65]. This cellular response was mediated through RhoA-GTPase signalling coupled with actin cytoskeletal reorganisation, and significantly, could be mimicked in vitro by culturing embryos in medium deficient in the BCAAs indicating this metabolite restriction to be the activator [63]. In recent studies, we show that of the three BCAAs, isoleucine deficiency is the most potent activator of TE histotrophic nutrition mediated through nuclear translocation of the transcription factor TFEB to drive lysosome biogenesis [65]. TFEB normally resides at the lysosome membrane associated with and phosphorylated by mTORC1 when nutrients are plentiful but, under conditions of nutrient deprivation, it becomes de-phosphorylated, uncouples and translocates to the nucleus to stimulate lysosomal gene expression [85,86].

Compensatory features of maternal Emb-LPD/LPD are maintained in post-implantation TE lineages. Ectoplacental cone explants (E8.5) display substantial expansion of the trophoblast giant cell (TGC) area over a 24–48 h culture in both LPD and Emb-LPD [87]. This adaptation was substantiated with enhanced spreading and proliferation of secondary TGCs emerging from LPD explants. Thus, the increased dynamics of placentation through motility and proliferation indicate improved functional efficiency from LPD and Emb-LPD treatments. Consequently, LPD- and Emb-LPD conceptuses in the perinatal period (E17.5) exhibit an increased foetal:placental weight ratio [87]. Similarly, maternal–foetal placental transport of glucose and AAs was increased by LPD treatment in late gestation, as was expression of relevant transporters, further supporting a sustained compensatory response [88].

Similar to the derivation and function of the placenta, the extraembryonic PE lineage, which generates the visceral yolk sac, shows evidence of altered phenotype following maternal Emb-LPD/LPD. The rodent yolk sac is a critical source of AAs for foetal growth mediated through endocytosis of maternal uterine nutrients by visceral endoderm cells and their transport into the vitelline circulation and the foetal compartment of the conceptus [80]. The early PE, studied in embryoid bodies derived from embryonic stem cell lines formed from Emb-LPD blastocysts, displays enhanced histotrophic nutrition, as seen in the TE with increased numbers and collective volume of lysosomes [63]. PE adaptation to the LPD environment coincides with reduced expression of GATA6, the principal transcription factor that regulates specification and differentiation of the PE through its downstream targets [89]. Reduced GATA6 expression is achieved through epigenetic regulation comprising histone deacetylation at the main *Gata6* promoter domain, which may allow increased proliferation prior to differentiation and histotrophic activity [89]. This cellular adaptation is maintained in the fully-formed visceral yolk sac at E17.5 with reduced GATA6 expression and increased expression of megalin receptor in visceral endoderm cells following Emb-LPD and LPD treatments, and with increased endocytosis capacity [40,89].

### 3.3. Blastocyst Nutrient Sensing Coordinates the Foetal Growth Trajectory

The compensatory responses of the extra-embryonic lineages in response to LPD and Emb-LPD discussed above will act positively to protect foetal growth despite poor maternal diet. The peri-conception induction of altered programming is also evident in the somatic tissues (e.g., liver and kidney) of the developing foetus. This regulation is primarily achieved through control of rRNA transcription during ribosome biogenesis (summarised in Figure 2c). rRNA, the structural backbone of the ribosome, is transcribed from tandem repeats of conserved rDNA genes organised in nucleoli. The ribosome is the cellular protein translation machine that controls growth across the whole organism and throughout life. Whilst there are several hundreds of rDNA gene copies available per cell, many are inactive physiologically through epigenetic mechanisms, and thus, the rate of transcription can be modulated by nutrient availability and other factors such as ageing [90,91,92]. rDNA silencing includes epigenetic regulation through hypermethylation of the rDNA promotor restricting Pol 1 binding and transcription, working alongside ribosome-associated proteins that coordinate Pol 1 activity and sense cellular environment [91,93].

In our Emb-LPD/LPD model, rRNA transcription rate is exquisitely regulated by maternal diet. When maternal LPD is sustained throughout gestation, rRNA transcription in foetal tissues is reduced compared with NPD controls; however, postnatally, after release from dietary protein restriction, rRNA expression increases to a level above that of controls. Moreover, following the transient maternal Emb-LPD, both foetal and adult tissues display rRNA expression at levels above that of controls [94]. This pattern of rRNA expression is regulated epigenetically; methylated DNA immunoprecipitation (MeDIP) assays revealed suppressed rRNA expression coincided with increased rDNA methylation and vice versa [94]. In addition, we find this maintained dietary responsiveness is mediated through the ribosome transcription factor, RRN3, a regulator and binding partner of Pol 1 activity in rRNA transcription sensitive to nutrient availability and which controls Pol 1 assembly at the rDNA promoter [95,96]. Thus, expression of RRN3 during foetal development, similar to that of rRNA, is enhanced by release from the dietary challenge, and RRN3 overexpression, as shown in culture cells, acts to coordinate ribosome biogenesis by rDNA promotor demethylation and increased rRNA expression [94].

This sensitive mechanism to coordinate maternal nutrient availability with ribosome biogenesis through rDNA methylation and rRNA expression has been confirmed in related mouse models and may also act in response to obesogenic diets [97]. These data first demonstrate that maternal diet experience before embryo implantation has a lifetime influence on ribosome biogenesis and growth, suppressing rRNA production when nutrients are restricted but stimulating rRNA expression beyond control levels when nutrient availability is restored (summarised in Figure 2c). Second, the mechanism gives new insight to the ‘thrifty phenotype’ concept, initially proposed in relation to risk of metabolic syndrome, whereby thrift in growth during nutrient adversity would predispose to disease in later life if nutrients became plentiful [98,99]. This mismatch anticipated ‘thrifty genes’ to enhance survival by promoting storage of nutrients when in excess, such as by fat deposition, a protective mechanism to withstand later nutrient deprivation [100,101]. The close association of rRNA production to concurrent nutrient availability in our model, its application across different somatic tissues and its lifetime reach originating from preimplantation experience make it a potent mechanism in DOHaD programming of growth control.

Lastly, we need to consider how environmental nutrient availability is perceived to regulate RRN3 expression and ribosome biogenesis. RRN3 expression is regulated by environmental factors, reduced by caloric and other nutrient restriction and sensitive to AA availability and mTORC1 signalling [91,92,102]. Indeed, PTEN, the negative regulator of mTORC1, has been proposed as a thrifty gene to drive metabolic storage when nutrients are plentiful [103]. We can, therefore, postulate a stepwise progression in the timeline of Emb-LPD/LPD programming from its induction in the embryo through mTORC1, discussed below.

### 3.4. From Peri-Conception Exposure to the Endgame of Disease Risk

We have seen a stepwise progression links peri-conception maternal Emb-LPD/LPD with adult disease risk in our mouse LPD model. We propose early embryos are active participants in environmental nutrient sensing to select a ‘strategy for development’, to optimise offspring fitness and survival with mTORC1 a coordinating signalling pathway. In summary, the protein restricted diet diminishes the bioavailability of AAs, specifically BCAAs, within the immediate micro-environment of the blastocyst. The embryo senses this deficiency through reduced mTORC1 signalling, which is used to activate the survival strategy in subsequent extra-embryonic and embryonic/foetal lineages. The combination of reduced BCAA (especially isoleucine) and reduced mTORC1 activates the mTORC1-associated TFEB to coordinate lysosome biogenesis and histotrophic nutrition. Other features of placental and yolk sac compensatory responses may be linked to this activation mechanism, since mTORC1 is a critical player in placental function and maternal-nutrient provision [104,105]. Likewise, in embryonic lineages, the mTORC1-responsive RRN3 coordinates rRNA transcription and ribosome biogenesis with nutrient availability, a response to control growth activated in the peri-conception period [94]. Critically, our data further implicate a link between mTORC1 and epigenetic modifications to mediate altered function, as seen in PE [89] and foetal tissues [94]. One new direction in mTORC1 investigation is its interaction with regulators of epigenetic organisation to modify chromatin structure to control gene expression [106]. We speculate that the epigenome reorganisation occurring in early embryogenesis to establish the new genome [5,6] may include a focus for mTORC1 to influence environmental developmental reprogramming.

How do these modifications in signalling and growth regulation mediated from peri-conception experience throughout gestation drive increased cardiometabolic and neurological disease risk in adulthood? Weight of neonates is positively correlated with adult weight and cardiovascular and neurological behaviour patterns in both Emb-LPD and LPD offspring [40]. Perinatal weight is well recognised to be predictive of adult chronic disease risk in many DOHaD models [107]. Comparison of Emb-LPD and LPD offspring nutritional experience pinpoints their specific maladaptation. Thus, from implantation, Emb-LPD embryos and foetuses experience sufficient (control) maternal diet coupled with compensatory placental and yolk sac nutrient delivery and enhanced ribosome biogenesis to drive foetal growth, a potent mix that leads to overgrowth from late gestation. In LPD offspring, the restrictive maternal diet throughout gestation and reduced rate of ribosome biogenesis temper potential maladaptation and reduce birthweight [40]. However, whilst reproductive fitness is maintained in both treatments, disease risk is increased in post-reproductive life. Indeed, the peri-conception responsiveness to maternal diet, critical in lifetime health, appears ‘hard-wired’ in that Emb-LPD blastocysts transferred to control foster dams still display increased foetal growth in late gestation [40].

Evidence suggests the peri-conception programming via mTORC1 signalling in the mouse diet model has clinical significance. Maternal diabetes in a rabbit model causes plasma and blastocoelic cavity BCAA concentrations to rise, which increases blastocyst mTORC1 signalling and altered expression of BCAA regulatory enzymes [108]. In human UF, BCAA concentrations vary according to diet [109] and a healthy Mediterranean diet improved blastocyst development within an ART randomised controlled trial [110]. In a separate study, a related peri-conception Mediterranean diet altered child epigenome and reduced risk of adverse neurological behaviour such as depression [111]. Lastly, we have shown recently that human blastocysts from mothers with high BMI demonstrate enhanced trophectoderm histotrophic nutrition and nuclear translocation of TFEB compared with blastocysts from mothers with normal BMI, indicating embryo sensitivity and responsiveness to maternal physiological condition [65].

## 4. From Diverse Peri-Conception Environment to a Convergent Adult Disease Risk

Mechanistic analysis of maternal obesity and overnutrition broadly mirrors that of undernutrition in that a stepwise progression can be traced from peri-conception through gestation in extra-embryonic and foetal compartments, leading to offspring cardiometabolic disease risk [1,2]. Thus, oocytes and early embryos accumulate excess lipids and metabolites that disturb mitochondrial function and potential [112,113,114,115]. Moreover, nutrient excess and embryo metabolic stress lead to adverse gestational growth, again with a central role for mTORC1 signalling in placental efficiency and foetal growth [116].

In the case of micronutrient and vitamin supplementation around the time of conception and developmental consequences affecting health, again some convergence of effects can be seen. Whilst micronutrient deficiencies or excess throughout gestation have enduring effects on a range of pregnancy pathologies affecting placental efficiency, foetal growth and system dysfunction [117], impacts mediated around conception are critical. Thus, vitamin D insufficiency in peri-conception has adverse effects on blastocyst implantation and subsequent immune system development and modulation [118]. Maternal vitamin B12 and methionine deficiency around conception in sheep leads to cardiovascular and metabolic dysfunction and obesity in male offspring, associated with enduring altered methylation of the epigenome [119] with similar findings in a rat model [120]. Methyl group deficiency affects 1-carbon metabolism pathways, and therefore, disturbs the epigenome directly, which can subsequently affect gene expression and physiology of many systems [67,121]. Thus, folic acid insufficiency, known to be a risk factor for neural tube defects [117], also causes increased tumorigeneses and inflammation in offspring when restricted in peri-conception [122]. Immediate effects of folic acid availability in maternal diet during preimplantation development include gene expression of key regulators of foetal and extra-embryonic cell lineages and blastocyst TE proliferation [123].

In a further direction, peri-conception exposure to environmental pollutants and endocrine-disrupting chemicals can similarly disturb the developmental programme with long-term health outcomes that show convergence with those mediated through maternal undernutrition. The peri-conception period is one that is vulnerable to pollutant exposures which may act in short-term ways to perturb fertility, epigenetic status and incidence of congenital abnormalities [124,125]. However, endocrine disruptors also mimic endogenous hormones, so-called obesogens, leading to metabolic and cardiovascular disease risk in adult offspring [126,127].

Lastly, convergence in adult offspring phenotype from peri-conception environment also occurs in response to IVF and assisted reproductive treatments (ART) [1,2]. However, whilst there is evidence of ART affecting human birth weight [128] and associating with adverse cardiometabolic health in offspring [129,130,131], there are many confounders that complicate interpretation, including parental infertility status and varied demographics, the introduction of improved protocols over time and differences between clinics and the relatively young age of offspring born to date. Moreover, while epigenetic abnormalities are detected in ART offspring [132], variations in DNA methylation, potentially symptomatic of later disease, may not be permanent and can resolve by adulthood [133].

Animal models of ART, therefore, provide the opportunity to examine specific treatment conditions without the above human confounders to determine peri-conception effects on adult health. Thus, embryo culture in mouse ART models associate with cardiometabolic dysfunction in adulthood [134,135]. Mouse models also permit more subtle analyses of specific treatments to evaluate health implications. As an example, current clinical ART practice favours culture to blastocyst rather than cleavage stage before transfer, enabling improved selection of viable embryos and better synchronisation with uterine environment maternal cycle. Moreover, this strategy does not appear to affect birth weight or perinatal outcomes [136,137], but long-term health implications of extended culture are unknown. However, a mouse model whereby direct comparison between IVF and culture to either cleavage or blastocyst stages before transfer has demonstrated poorer cardiovascular but improved metabolic health from long culture [138].

## 5. Conclusions and Thinking Ahead

From our mouse model, we show that maternal undernutrition can be detected by the preimplantation embryo through nutrient-sensing signalling pathways which disturb metabolic homeostasis. This event, in turn, activates responses to control nutrient supply and aid survival involving extra-embryonic and embryonic lineages and persisting throughout gestation. Responses appear coordinated primarily through mTORC1 signalling but recruit a variety of mechanisms notable epigenetic and cellular to be accomplished. Whilst fitness and survival may be achieved, disease risk in later life becomes a trade-off. We see a similar pattern of stepwise responsiveness from peri-conception experience across diverse environments including maternal overnutrition and ART. How should we move forward from our current level of understanding of peri-conception DOHaD?

One major drawback of embryo models is the limited amount of cellular material available at the time of induction, a biomass of some 30–40 cells. Technologies for unbiased and integrated multi-omics across transcriptome, epigenome, proteome and metabolome analysis of single or small numbers of cells are advancing and suited to early embryo screening for DOHaD candidates [139,140,141]. Omics technologies are required for assessing human embryo competence in relation to diverse environments, such as culture composition [142,143,144], and also across time/maturation of the embryo [145].

An alternative approach to overcome the cellular limitation of early embryos in peri-conception DOHaD studies is to use embryonic stem (ES) cell lines expanded from blastocysts either in undifferentiated or differentiated (e.g., embryoid bodies) state. This latter approach has been shown to be successful in our mouse Emb-LPD model for characterising PE histotrophic nutrition and epigenetic effects [63,89]. Recently, we have shown derivation of mouse ES cells can be used in undifferentiated state to assess chromatin and expression changes mediated through advanced maternal age [146]. However, this approach may be limited by the stability of programming changes during derivation or after increasing passage number [147].

From a clinical perspective, the demonstration that the peri-conception period can be vulnerable to environment with lifelong health implications argues for preparation for pregnancy to include maternal health and diet before conception occurs. It is now becoming clear that randomised trials of maternal nutritional interventions that initiate after pregnancy is established, usually after the first trimester, are unsuitably timed, missing the peri-conception window [148,149,150,151]. Preconception interventions with power for child health follow-up offer improved opportunities for success, for example in child growth [152,153]. Moreover, preconception supplementation also influenced the epigenome of children up to 9 years [154]. These new and emerging datasets authenticate the susceptibility of peri-conception environment from experimental research, and in turn, require new thinking on the social and educational policies to mediate key messages to adolescent women [155].
